# Bioactive Phenolic Compounds From Agri-Food Wastes: An Update on Green and Sustainable Extraction Methodologies

**DOI:** 10.3389/fnut.2020.00060

**Published:** 2020-05-07

**Authors:** Lucia Panzella, Federica Moccia, Rita Nasti, Stefania Marzorati, Luisella Verotta, Alessandra Napolitano

**Affiliations:** ^1^Department of Chemical Sciences, University of Naples “Federico II”, Naples, Italy; ^2^Department of Environmental Science and Policy, Università degli Studi di Milano, Milan, Italy

**Keywords:** phenolic compounds, agri-food wastes, sustainability, microwave assisted extraction, ultrasound assisted extraction, supercritical fluid extraction, deep eutectic solvents, Naviglio extractor

## Abstract

Phenolic compounds are broadly represented in plant kingdom, and their occurrence in easily accessible low-cost sources like wastes from agri-food processing have led in the last decade to an increase of interest in their recovery and further exploitation. Indeed, most of these compounds are endowed with beneficial properties to human health (e.g., in the prevention of cancer and cardiovascular diseases), that may be largely ascribed to their potent antioxidant and scavenging activity against reactive oxygen species generated in settings of oxidative stress and responsible for the onset of several inflammatory and degenerative diseases. Apart from their use as food supplements or as additives in functional foods, natural phenolic compounds have become increasingly attractive also from a technological point of view, due to their possible exploitation in materials science. Several extraction methodologies have been reported for the recovery of phenolic compounds from agri-food wastes mostly based on the use of organic solvents such as methanol, ethanol, or acetone. However, there is an increasing need for green and sustainable approaches leading to phenolic-rich extracts with low environmental impact. This review addresses the most promising and innovative methodologies for the recovery of functional phenolic compounds from waste materials that have appeared in the recent literature. In particular, extraction procedures based on the use of green technologies (supercritical fluid, microwaves, ultrasounds) as well as of green solvents such as deep eutectic solvents (DES) are surveyed.

## Introduction

Global food waste approximates 1.3 billion tons per year as the result of primary and secondary processes occurring along the supply chain, which include losses generated during production and postharvest of the food products, that represent about 75% of food losses e.g., in developing African countries, or wastage at the consumption stage as is the case of industrialized countries (North America and Europe) (1, 2). Agri-food industry in particular is responsible for the generation of high volumes of organic wastes (biomasses), reaching up to 140 billion tons per year, although a considerable part of this is not related to food wastage issues (2–4). Disposal of these byproducts represents a cost to the food processor and has a negative impact on the environment. On the other hand these materials can be considered as a largely available, low cost source not only of energy for biofuel production, but also of value-added compounds, whose recovery represents therefore a valuable opportunity (5).

Generally, natural products are considered attractive value-added compounds based on their wide bioactivity spectrum. Among these, a prominent role is occupied by phenolic compounds, which are well-known for their beneficial effects on human health, e.g., in the prevention of cancer and cardiovascular diseases (6–8). These effects have been ascribed in part to their ability to act as potent antioxidants and scavengers of reactive oxygen species, generated under oxidative stress conditions and responsible for the onset of several inflammatory and degenerative diseases (9–11). These properties have therefore prompted the use of natural phenolic compounds not only as food supplements (7, 12–15), but also as additives for functionalization of materials to be used e.g., in biomedicine (16–18), cosmetic (19–22), or food industry (23–27).

In this context of course it is clear that, in order to comply with the principles of the green economy, the recovery of phenolic compounds from agri-food wastes should be achieved using environmentally friendly, sustainable and possibly low-cost procedures. On this basis, this review will provide an overview of the most commonly employed green approaches for the recovery of functional phenolic compounds from agri-food byproducts. In particular microwave assisted extraction (MAE), ultrasound assisted extraction (UAE), and supercritical fluid extraction (SFE) have been considered as well as the use of deep eutectic solvents (DES) as emerging green solvents. A brief description of other promising sustainable methodologies based e.g., on the use of Naviglio extractor®, pulse electric fields (PEF) and steam explosion will also be provided. Patents were excluded since the main aim of this review is an update of those applications that have a potential for further development but may not be ready for a straightforward use in industries.

## Phenol-Rich Agri-Food Wastes

### Fruit Byproducts

#### Grape and Wine Byproducts

**Figure 1:**
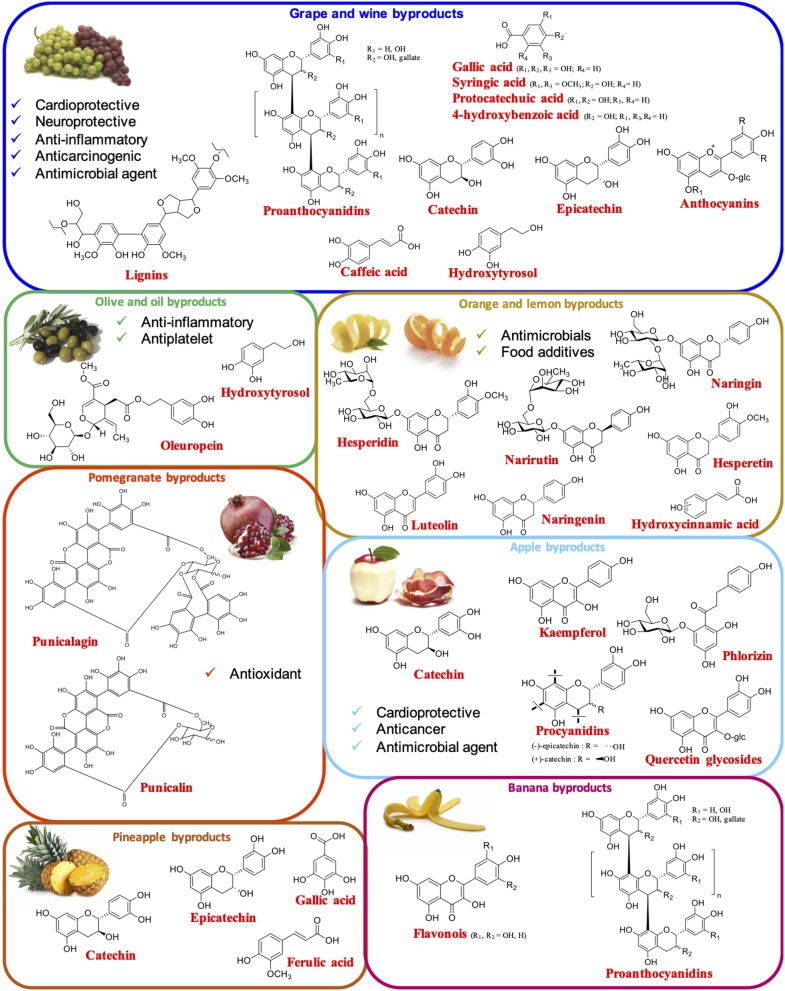
Main fruit byproducts and their most prominent phenolic constituents with reported bioactivities.

The main byproduct of the wine industry is known as grape pomace and consists mainly of grape skin, seeds, stems, and remaining pulp (28). Approximately 9 million tons of this waste are produced per year in the world, which represents about 20% w/w of the total grapes used for wine production (29, 30). As to the phenolic composition, an average lignin content of 17–24% w/w has been reported (31). Condensed tannins (proanthocyanidins) represent another main class of polyphenols present in the pomace, together with other small phenolic compounds exhibiting high health beneficial properties, such as cardioprotective, neuroprotective, anti-inflammatory, anticarcinogenic, and antimicrobial activities. Among these, the most abundant are phenolic acids (caffeic, gallic, protocatechuic, 4-hydroxybenzoic, and syringic acid), hydroxytyrosol, and flavonoids, mainly catechin and epicatechin derivatives as well as anthocyanins, which are commonly recovered and used as food colorants (28) (Figure 1).

#### Olive and Oil Byproducts

The olive oil industry also generates high amounts of byproducts, which are particularly rich in lignans, secoiridoids, and especially hydroxytyrosol, which is one of the most bioactive phenolic compounds present in nature, endowed with anti-inflammatory and antiplatelet properties (12, 28–30, 32) (Figure 1). Soybean (33) and palm oil (34) byproducts have been also described as a valuable source of polyphenols.

#### Orange and Lemon Byproducts

Citrus peels as well as seeds and pulp deriving from the industrial production of orange and lemon juice, which led to about 15 million tons of waste per year, are an important source of hydroxycinnamic acids and flavonoids, mainly flavanone glycosides (hesperidin, naringin, and narirestin), flavanones (hesperetin and naringenin), and flavone aglycons (luteolin) (28, 30, 35) (Figure 1). Extracts rich in these compounds have been proposed to be used as antimicrobials or as food additives to impart bitter taste to food and beverages (30).

#### Pomegranate Byproducts

As in the case of citrus fruits, pomegranate juice production leads to the generation of high amounts of wastes (ca. 9 tons for 1 ton of juice) (36), containing very specific compounds such as the ellagitannins punicalagin and punicalin, which are endowed with very high antioxidant potency (17, 30, 37) (Figure 1).

#### Apple Byproducts

Also apple pomace represents an important source of valuable polyphenols, exhibiting antimicrobial, anticancer, and cardioprotective activities. Among these a prominent role is played by quercetin glycosides, kaempferol, catechin, procyanidins, and especially the dihydrochalcone phlorizin (28, 30, 38–40) (Figure 1).

#### Other Fruit Byproducts

Banana peels contain high amounts of phenolic compounds, particularly flavonoids and proanthocyanidins (28, 41), whereas pineapple peels are a source of gallic acid, catechin, epicatechin, and ferulic acid (28, 42) (Figure 1). Also different nut shells as well as endocarps and skins of berries (43), apricot (44), acerola (45), xonocostle (46), litchi (47), sea buckthorn (48), pequi (49, 50), juçara (50), and dragon fruit (51) are emerging as valuable sources of phenolic compounds. Tea residues also lead to phenolic-rich extract (52, 53).

### Vegetable byproducts

#### Onion Byproducts

**Figure 2:**
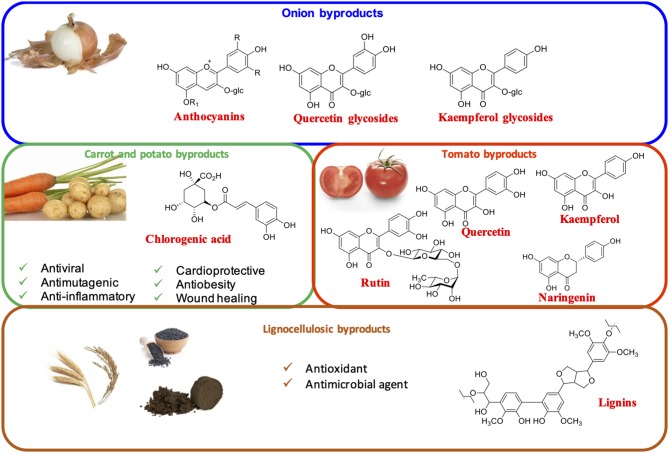
Main vegetable and lignocellulosic byproducts. Shown are the most abundant phenolic components and the reported bioactivities.

The major byproduct resulting from industrial peeling of onions is represented by the skin, the outer fleshy leaves, and the top and bottom bulbs, which are produced in more than 450,000 tons only in Europe (30, 54). These are particularly rich in flavonoids such as quercetin and kaempferol glycosides. Anthocyanins are also present in red onions (Figure 2) (28, 55).

#### Carrot Byproducts

The main carrot byproduct is the pomace deriving from carrot juice production. This is rich in hydroxycinnamic acid derivatives, particularly chlorogenic acid, which are known to possess antiviral, antimutagenic, anti-inflammatory, cardioprotective, antiobesity, and wound healing properties (Figure 2) (28, 56).

#### Potato Byproducts

Potato peels are undoubtedly among the most abundantly produced vegetable byproducts. Their extracts have been proposed for several applications in the food and other sectors. The main phenolic compounds present in potato peels are phenolic acids and derivatives, especially chlorogenic acids (Figure 2) (28).

#### Tomato Byproducts

Peels and seeds from tomato processing contain mainly flavanones (naringenin glycosylated derivatives) and flavonols, mainly quercetin, rutin, and kaempferol glycoside derivatives (Figure 2) (28, 57).

#### Other Vegetable Byproducts

Other vegetables that lead to high amounts of byproducts are fennels (58, 59), broccoli (60), cabbages (61), lettuce (62, 63) and artichokes (64, 65).

### Lignocellulosic Byproducts

Lignocellulosic agri-food byproducts such as wheat straw (28, 30), wheat bran (66) and distiller's grain (67), spent coffee grounds from the industrial production of soluble coffee (68, 69), sawdust (70, 71) and other wastes from the wood industry (72) have been widely described as a clean source of phenolic compounds, mainly deriving from hydrothermal and/or autohydrolysis processing of lignin, that could be exploited for application in a variety of sectors given their antioxidant and antimicrobial properties (28) (Figure 2).

## Green Extraction Techniques

### Microwave Assisted Extraction (MAE)

**Figure 3:**
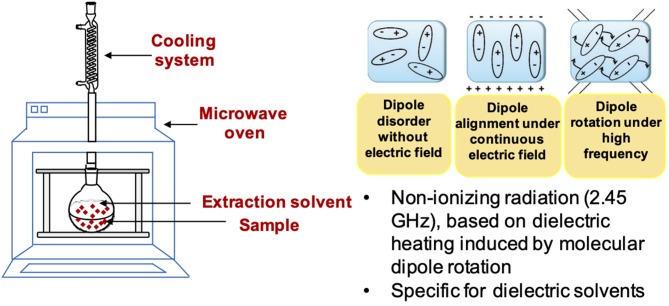
Schematic representation of MAE equipment and characteristics.

Microwave assisted extraction (MAE) can be classified as a green extraction technique since it shortens the extraction time and reduces the consumption of solvent. The principle on which MAE is based is the dielectric heating, that is the process in which a microwave electromagnetic radiation heats a dielectric material by molecular dipole rotation of the polar components present in the matrix (73) (Figure 3). MAE has been reported to proceed through several distinct steps as the result of heat and mass gradients generated into the matrix: (1) penetration of the solvent into the matrix; (2) solubilization and/or breakdown of the components; (3) transport of the solubilized compounds from the insoluble matrix to the bulk solution; (4) separation of the liquid and residual solid phase (74, 75). Several parameters should be considered to optimize the MAE process, that is solvent, solid to solvent ratio, microwave power and extraction temperature and time. As to the solvent, ethanol, alone or in combination with water, is one of the most commonly used in MAE because it has a good capacity to absorb the microwave energy and exhibits good solubilizing properties toward phenolic compounds. The amount of solvent to be used has to be properly chosen to ensure complete immersion of the sample during the entire irradiation process, avoiding excessive amounts that would require time and energy consumption for removal in the final recovery of the extracted compounds. The choice of the microwave power as well as of extraction temperature and time is dependent on the stability of the compounds to be extracted (75).

Other factors can also affect the efficiency of the extraction, such as the characteristics of the matrix in terms of particle size, the contact surface area and the water content. As an example, higher extraction yields of phenolics can be achieved by milling the sample into smaller particle sizes, although particles smaller than 250 μm can be difficult to separate from the liquid phase at the end of the process (75).

Regarding the instrumental apparatus, MAE can be performed in closed extraction containers, which operate at high pressures and temperatures, allowing for higher extraction yields, or in open vessels operating under milder conditions, at atmospheric pressure. This latter system is particularly suitable for thermolabile compounds and has the advantage of requiring a low-cost instrumentation able to process higher amounts of material. Recently, instruments operating under vacuum or under a nitrogen atmosphere have also been developed (75).

### Ultrasound Assisted Extraction (UAE)

**Figure 4:**
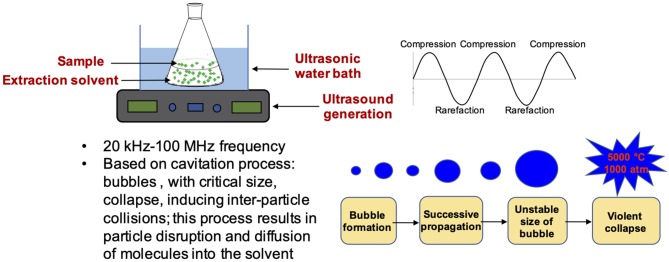
Schematic representation of UAE equipment and characteristics.

As in the case of MAE, ultrasound assisted extraction (UAE) also allows to reduce the time and solvent amount needed to efficiently extract phenolic compounds from agri-food wastes. UAE is considered one of the simplest extraction procedure since it requires common laboratory equipment, that is an ultrasonic bath (76). The technique is based on the cavitation process induced by compression and expansion cycles associated to the passage of ultrasounds (20 kHz-100 MHz frequency) through the sample. The implosion of the cavitation bubbles induces inter-particle collisions which result, among others, in particle disruption and enhanced diffusion of the extractable compounds into the solvent (Figure 4). Sample characteristics such as consistency, rheology and particle mobility can therefore significantly affect the ultrasound energy dispersion and hence the effectiveness of UAE.

UAE is generally performed under static conditions, that is in closed vessels, with no solvent refreshing, or in a dynamic mode, in which fresh solvent is supplied in continuously (75).

### Supercritical Fluid Extraction (SFE)

**Figure 5:**
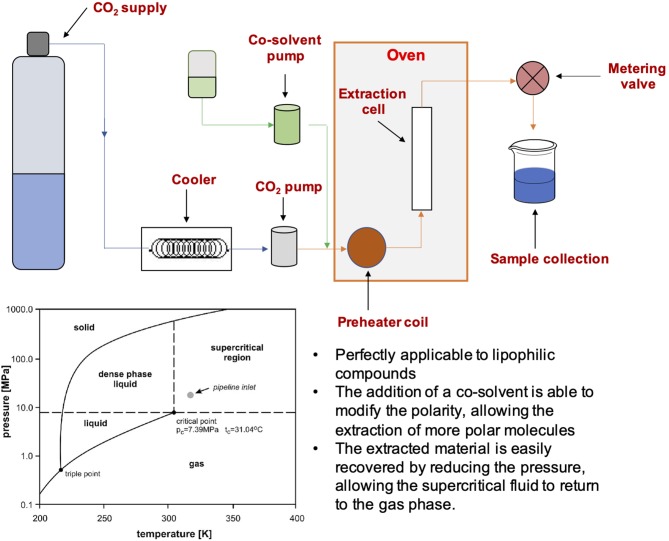
Schematic representation of SFE equipment and characteristics.

Another green technology, based on supercritical CO_2_ (scCO_2_), has been recently considered in order to overcome the environmental concerns related to conventional methods. scCO_2_ is in fact characterized by immediate advantages over traditional solvent-based methods. It enables the selective extraction of compounds soluble in scCO_2_, thus perfectly applicable to lipophilic compounds like fats, with no need of concentration steps (77). The addition of a co-solvent (for example ethanol, that is well-tolerated by various industrial sectors) is able to modify the polarity of the scCO_2_ allowing the extraction of more polar molecules (78) (Figure 5). Moreover, the operative temperatures can be set low enough to avoid the degradation of thermolabile substances. Literature results show a substantial advantage with respect to conventional extraction in terms of easy recovery, selectivity, compounds stability, time, and an overall total energy saving (79).

The high versatility of SFE technique can be extended to industrial scale with the intent to introduce sustainability to large-scale processes. Moreover, the easy removal of CO_2_ at ambient conditions and its feasible recovery through specific apparatus for its reuse lead to a reduction of reagent-related costs, well-appreciated in the industrial sector.

A relevant aspect in SFE, affecting the extraction rate, is the solubility of target compounds in the scCO_2_. In this case, temperature and pressure are key thermodynamic parameters that mainly contribute to solubility of target compounds. In details, the increase of pressure enhances the density of supercritical fluid and its solvation power (80). Working on vegetal matrix, high pressure can disrupt plant cell thus facilitating the release and the solubilization of compounds. Temperature has a more complex role: at constant pressure, its increase enhances the vapor pressure of the solute and its solubility in the extractor fluid with a slight but balanced decrease of supercritical fluid density.

Many examples in literature show how the scCO2 has been widely applied to lipophilic molecules extraction (81). Conversely, there are fewer applications on target molecules of higher polarity when the addition of a co-solvent to scCO_2_ is necessary to enable their extraction. The use of co-solvents affects the physical and chemical intermolecular forces of the system and increases the local density around a solute molecule, achieving specific interactions such as H-bond.

Some works in the literature recently reported the recovery of polyphenols from wastes through scCO_2_. Bioactive and valuable compounds isolation from agri-food residues by green technologies like scCO_2_ is of particular interest because it accomplishes the implementation of bio-based-economy policies.

Overall, not only the physico-chemical parameters of scCO_2_ extraction (temperature, pressure, and amount of co-solvent), but also the biomass nature and processing before extraction (lyophilization, micronization, etc.) deeply affect the final extraction yields and composition, being the diffusivity inside the solid matrix a critical parameter. The process conditions in SFE may differ from one matrix to another, even in the presence of the same compounds (82). The literature in the field is not as wide as in the case of the other green techniques, at least when scCO_2_ is applied to extract more polar molecules. The optimization of extraction parameters, such as pressure, temperature and the percentage of modifier, together with the understanding of matrix effects, are the key points to yield more polar molecules, but a wide literature sink to be set as background is still missing in this field.

### Deep Eutectic Solvent (DES) Extraction

Solid-liquid extraction is one of the most commonly used procedures to extract phenolic compounds e.g., from agri-food wastes (83). However, this methodology typically involves long extraction time periods, high costs, low yields, and the use of organic solvents, which even if exhibit excellent ability in phenolic compound dissolution and extraction, show many intrinsic drawbacks, such as low boiling points, flammability, toxicity, and non-biodegradability (84, 85). On the other hand, water is as an extraction solvent effective only for polar and hydrophilic compounds (86, 87). Therefore, there is a high demand for green solvents exhibiting the same excellent extraction properties of organic solvents, but low-costs and minimal environmental impact (87, 88).

Recently, a new type of eco-friendly and green solvents called deep eutectic solvent (DES) has been developed and applied in the extraction of phenolic compounds (87–90).

**Figure 6:**
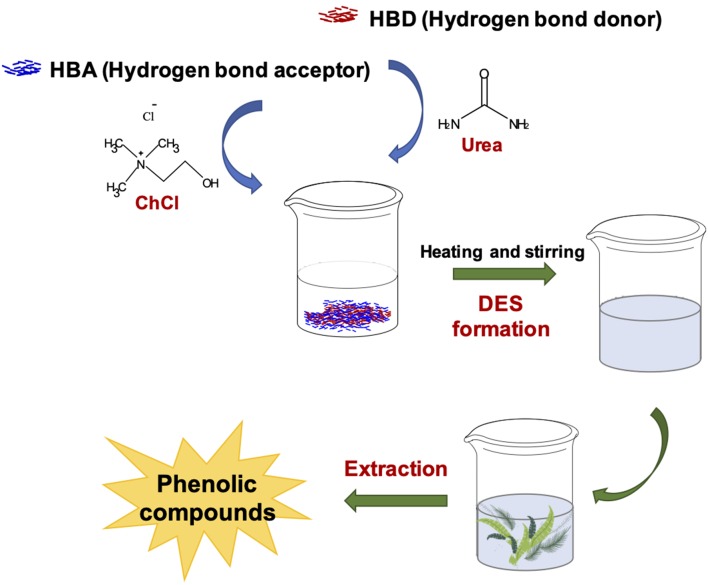
Schematic representation of extraction of phenolic compounds with DES.

DES preparation was first described by Abbott et al. (91). They are easily prepared by mixing, at a suitable temperature, a hydrogen bond acceptor (HBA) and a hydrogen bond donor (HBD) (91) (Figure 6). Compared to common organic solvents, DES offer many advantages such as low price, easy preparation, and easy availability. Moreover, most of them are biodegradable with very low toxicity (90, 92).

DES can be described by the general formula Cat^+^X^−^zY, where Cat^+^ is typically ammonium, sulfonium, or phosphonium, X^−^ a Lewis base, normally a halide, Y a Lewis or Brønsted acid, that forms a “complex” with X^−^, and z is the number of Y molecules that interact with the anion (93). These interactions result in the formation of a eutectic mixture, characterized by a melting point lower than that of individual constituents.

The most popular component used for the preparation of DES is choline chloride (ChCl), a cheap and non-toxic salt. The most used HBD are urea, ethylene glycol, glycerol, but also alcohols, amino acids, carboxylic acids and sugars (94, 95). Indeed, very recently, DES have been developed from the combination of primary metabolites and bio-renewable starting materials. These solvents have been called “natural deep eutectic solvents” and have been obtained by combining compounds abundantly present in nature that play important roles for solubilizing, storing or transporting metabolites in living cells and organisms (96, 97).

The physicochemical characteristics of DES, such as freezing point, conductivity, density, viscosity and polarity, normally depend on their composition, therefore it is possible to modulate them by modifying the HBD and HBA components. Generally the densities of DES are higher than water, and higher than the individual components (98). Also the viscosity of most DES is high (> 100 cP) at room temperature (89) as the results of the hydrogen bond network between the components leading to a lower mobility of the species. The large ion size and the electrostatic or van der Waals interactions between the components may also contribute to the high viscosity of DES. The conductivity of DES is generally poor, due to their high viscosity.

The ability of DES of donating and accepting protons and electrons as well as to form hydrogen bonds confers them good dissolution properties toward phenolic compounds, as recently explored also in the case of agri-food wastes.

## Application of Green Extraction Techniques to Phenol-Rich Agri-Food Wastes

### Grape and Wine Byproducts

**Figure 7:**
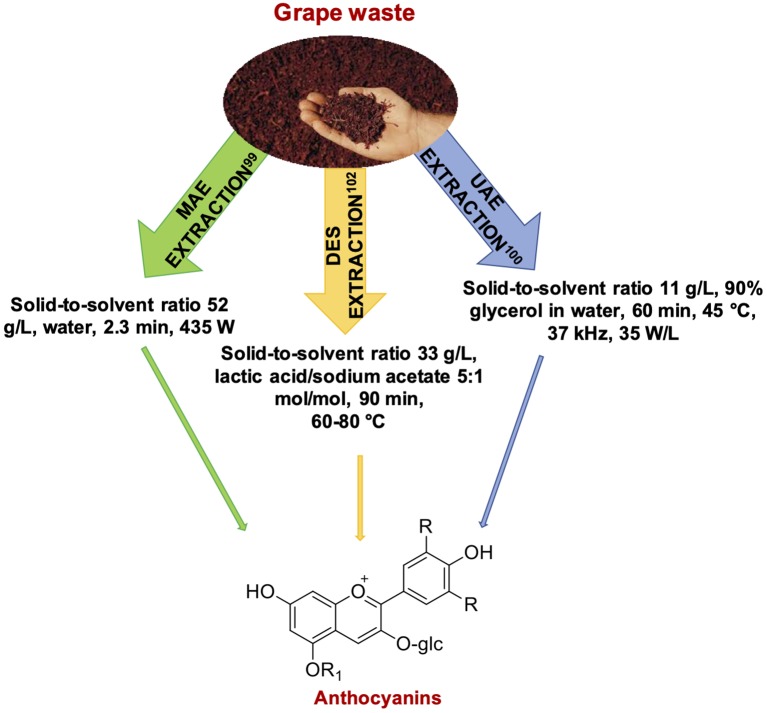
Representative examples of phenolic compounds recovered from grape byproducts.

Anthocyanins undoubtedly represent one of the main class of phenolic compounds recovered from grape-processing byproducts. Response surface methodology (RSM) coupled with genetic algorithm allowed to determine the optimal MAE conditions for the recovery of these pigments from grape juice waste. These were microwave power of 435 W, exposure time of 2.3 min and solid to solvent (water) ratio of 52 g/L. Under these conditions an anthocyanin yield of *ca*. 1.3 mg/g was obtained (99) (Figure 7). Anthocyanins together with other polyphenols have been efficiently extracted from winery byproducts also by UAE, requiring however the use of glycerol (90% w/v in water) as solvent and a lower (11 g/L) solid to solvent ratio (100) (Figure 7). Ten different ChCl-based DES have been also comparatively evaluated as solvents for anthocyanin extraction from grape pomace, and the highest efficiency was found for ChCl-citric acid. On these basis, new citric acid-based DES were prepared, and citric acid/maltose 4:1 molar ratio led to a significantly higher total anthocyanin content (TAC) when compared to reference solvents, particularly when combined with UAE (101). A DES composed of lactic acid-sodium acetate at a molar ratio of 5:1 has also been found efficient for pigment extraction from red grape pomace (Figure 7), whereas a 5:1 glycerol-sodium acetate mixture performed better for flavonoid extraction (102). A significant improvement in anthocyanin extraction yields from wine lees compared to acidified aqueous ethanol has been reported using ChCl-malic acid containing 35% v/v water combined with UAE (extraction time, 30.6 min; ultrasound power, 341.5 W) (103).

Resveratrol represents another important bioactive phenolic compounds which has been the focus of several studies directed to the optimization of the better conditions allowing for its efficient extraction from grape and wine byproducts. As an example, orthogonal test indicated a material to ethanol ratio of 50 g/L, an extraction time of 30 min, an extraction temperature of 55°C and a microwave power of 1.0 kW as the best conditions for MAE of resveratrol from grape pomace (104). Yields of about 30 mg per 100 g of dried extract were instead reported from Pinot noir seeds by performing MAE at 60 W for 30 min, using methanol as solvent with a solid to liquid ratio of 200 g/L (105). A more energy-efficient process for resveratrol recovery from red grape wastes has been reported by means of UAE using polyethylene glycol (PEG) as a co-solvent, allowing for lowering the amount of ethanol used in the extraction process. The optimized conditions as determined from RSM-Box-Behnken design involved a combination of 19 min, 54°C, and an ethanol/PEG/water ratio of 48:32:20 v/v/v (106). Likewise, a 1.5% aqueous β-cyclodextrin solution showed to be an excellent UAE medium for grapevine waste (107).

Phenolic compounds have been extracted also from grape skins. Very short extraction times (83 s, with a microwave power of 900 W) have been reported for the MAE of phenolic compounds from grape skins (108). Longer extraction times (50 min, at 65°C, with a solid to liquid ratio of 100 g/L) have been instead reported in the case of UAE using ChCl-oxalic acid as DES in presence of 25% water (109). Promising antioxidant and antiproliferative activity against cancer cells have been described also for a ChCl-malic acid phenolic extract obtained from grape skin (110).

Based on what reported above, it can be concluded that MAE generally requires short extraction times compared to the other techniques, although the use of more innovative methodologies, based e.g., on the use of DES, seem not to have been fully explored yet. Moreover, compared to the other widely exploited green methodology, that is UAE, a survey of the literature revealed MAE to be the first choice for the recovery of phenolic compounds from grape-derived wastes as summarized in the following.

MAE has been reported to be particularly effective in the case of vine shoots from Portuguese grapes. A total phenol content (TPC) of 32 mg gallic acid equivalents (GAE)/g was obtained by extracting dried vine shoots (0.1 g) at solid to solvent ratio of 5 g/L with ethanol: water 6:4 v/v for 20 min at 100°C. The extract thus obtained was significantly more effective than ascorbic acid in protecting erythrocytes against 2,2'-azobis(2-amidinopropane)-induced hemolysis. Moreover, it exhibited quite low IC_50_ values as inhibitor of acetylcholinesterase (IC_50_: 17–25 μg/mL) and α-amylase (IC_50_: 60–74 μg/mL) and presented promising antibacterial and antifungal activity. HPLC analysis indicated gallic acid, catechin, myricetin and kaempferol-3-O-rutinoside as the major contributors to the observed biological activities (111).

Another study also reported ethanol MAE extraction as an effective technique to obtain a polyphenol-rich, antioxidant extract from grapevine shoots. A plant/solvent ratio of 100 g/L was used, at 60°C for 30 min, with a 1.5 kW microwave power, under a 5 bar nitrogen pressure. The same apparatus but using acetone/water 8:2 v/v as the solvent, for 49 min, has been described also for the recovery of polyphenols from hazelnut skins (107).

Grape marc was also found to provide the extract with the highest TPC (143 mg GAE/100 mL) and highest antioxidant properties (239 mmol and 1,145 mmol of Trolox eqs/100 mL from the 2,2-diphenyl-1-picrylhydrazyl (DPPH) and 2,2'-azino-bis(3-ethylbenzthiazoline-6-sulfonic acid) (ABTS) assay, respectively) when MAE was applied to a series of agri-food wastes such as chicory, cabbage, celery, fennel, olive leaf, and grape marc wastes. The extraction was performed at 750 W for 4 min with water, using a solid to liquid ratio of 1,000 g/L. The obtained aqueous extracts were used as water substitute in dough formation to fortify bread, with the grape marc extract conferring food antioxidant activities to both the crust and crumb of bread (112).

Also red wine lees have been described as an important source of polyphenols, using a combination of MAE and membrane-based filtration (113).

Very recently, microwave pretreatment prior to conventional solid-liquid extraction has been found to lead to overall better outcomes for the preparation of polyphenol-rich extracts from winemaking process wastes with cosmeceutical potential (114). High efficiency has been reported also for ultrasound-assisted emulsification-extraction of polyphenols from grape seeds and alperujo, using methanol/water (dispersed phase)-hexane (continuous phase) emulsions formed in the presence of ultrasounds (115). Other sustainable UAE treatments have also been described in the case of grape pomace (116).

The possibility to recover high-value polyphenols by SFE starting from skin and seed fraction of grape pomace has been also investigated (117), comparing the results of conventional and not-conventional extraction. In this work, the temperature and pressure range were 40–60°C and 350–500 bar, respectively. The results confirmed that the final compositions (not reported) of the extract obtained through supercritical and conventional methods were similar, but scCO_2_ was more selective. In agreement with the literature, results showed that extraction of polyphenols was possible only after the addition of ethanol as co-solvent. In this case, however, co-solvent amounts >5% do not significantly affect the extraction yield. The authors hypothesized that high concentration of ethanol in the scCO_2_ enhances the formation of strong H-bond between the solvent and the solute. A second speculation concerns the possibility that some polyphenols could be solubilized by the adsorbed-ethanol molecules remaining entrapped in the solid matrix at the end of the SFE process.

### Olive and Oil Byproducts

Differently from what reported above for grape-derived wastes, DES has been widely applied to the extraction of phenolic compounds from olive and oil byproducts, combined in some cases with MAE or UAE.

**Figure 8:**
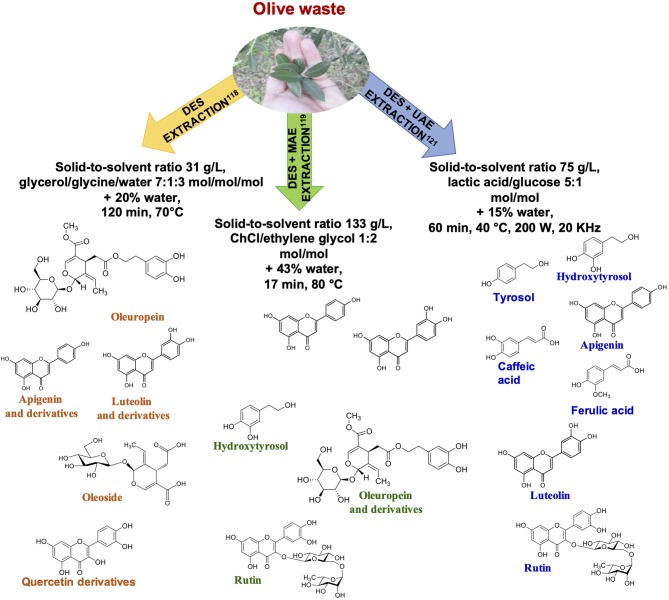
Representative examples of phenolic compounds recovered from olive byproducts.

Polyphenol extraction from *Olea europaea* leaves have been reported using glycerol-glycine-water 7:1:3 molar ratio. Optimized parameters in terms of total polyphenol yield and antioxidant power were 80% in water (w/w) DES concentration and a solid to liquid ratio of 31 g/L, at 70°C. Under these conditions a 18–30% higher total polyphenol yield was obtained compared to 60% aqueous ethanol, aqueous methanol and water, used as reference solvents. Furthermore, the DES extract exhibited significantly higher antiradical activity and reducing power (118) (Figure 8).

The use of different DES prepared from ChCl as HBA combined with MAE has been also reported for the extraction of phenolic compounds from olive leaves. RSM optimized extraction conditions were found to be 80°C and 17 min temperature and irradiation time, respectively, using 43% of water (119) (Figure 8).

Four different DES consisting of ChCl combined with maltose, glycerol, citric, and lactic acid in 1:2 molar ratio, 20% (v/v) of water, at 60°C have been proposed for the MAE of polyphenols from olive kernel and leaves. The best results were obtained with lactic acid based-DES, leading to the highest TPC (120).

Lactic acid-glucose 5:1 mol/mol implemented with 15% of water has also been proposed as a solvent for extraction of phenolic compounds from different byproducts of olive oil industry, combined with 30–60 min UAE at 40°C, using a solid-to-solvent ratio of 75 g/L (121) (Figure 8).

Recently, a blend of lactic acid/ammonium acetate 7:1 molar ratio with β-cyclodextrin (β-CD) has been used to recover polyphenols from olive leaves. The RSM optimized extraction conditions were: stirring speed 300 rpm, DES concentration in water 56% (w/w), solid to liquid ratio 10 g/L and β-CD concentration 0.7% (w/v). Maximum extraction yield was achieved at 80°C, without compromising antioxidant activity. Comparative assessment of the DES/β-CD extraction medium with other green solvents showed that it was a high-performing system providing polyphenol-enriched extract with improved antioxidant characteristics (122).

A relatively few number of papers have reported the UAE of phenols from olive wastes: these include for example recovery of polyphenols from industrial wastes of olive oil production such as olive tree leaves (123), or the obtainment of a phenolic yield of 45 mg/g for a virgin olive oil waste extract under RSM-determined optimum conditions, that is water:methanol 1:1 v/v, 60°C, 21 min (124). Ultrasound assisted enzymatic hydrolysis has also been established for extraction of phenolics from olive waste (125).

Similarly to grape- and wine-derived byproducts, also in this case shorter extraction times and higher efficiencies were obtained by use of MAE compared to conventional extraction methodologies.

Higher amounts of hydroxytyrosol (1.2 g/kg) and higher DNA strand scission inhibition activity compared to conventional extracts were found following MAE of olive pomace using power of 700 W over 10 min in a closed vessel system and 20% ethanol as the solvent (126).

Microwave irradiation has been combined with enzymatic hydrolysis to enhance the recovery of phenolic compounds also from palm oil mill effluents. Ragi tapai, a traditional fermented asian food, was used as the enzyme source, and MAE was performed at a solid to liquid ratio of 50 g/L for 4–5 min, with a microwave power of 180 W, that is low enough to avoid enzyme denaturation. The best results were obtained using 50% ethanol as the solvent, leading to a more than 30% increase in polyphenol extraction yield compared to conventional maceration extraction (127).

The advantages of MAE over conventional extraction techniques in terms of extraction times have been highlighted also for the recovery of isoflavones from soybean processing byproducts. In this case a 187.5 W power was applied for 3 min, using 80% ethanol at a sample to solvent ratio of 40 g/L (128).

### Orange and Lemon Byproducts

**Figure 9:**
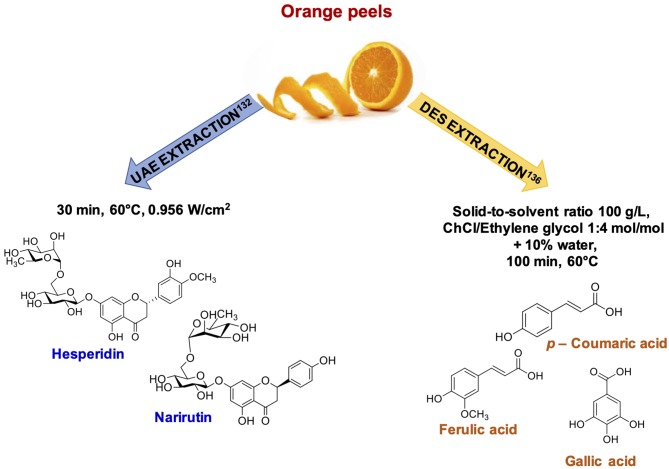
Representative examples of phenolic compounds recovered from orange byproducts.

Citrus byproducts seem to represent the most promising agri-food waste for the exploitation of UAE (129, 130). For example, a higher efficiency compared to MAE has been reported for the recovery of phenolic compounds from lime peel waste (131).UAE proved effective also in the case of orange peels, increasing the TPC yield by 30% compared to conventional extraction; statistical analysis revealed that the optimized conditions of ultrasound power and temperature were 0.956 W/cm^2^ and *ca*. 60°C, giving a polyphenol yield of *ca*. 50 mg GAE/100 g of dry matter (132) (Figure 9).

In another study a systematic evaluation of UAE parameters, including particle size, extraction time, extraction temperature and ultrasonic power for the recovery of *p*-coumaric acid, caffeic acid, chlorogenic acids, and hesperidin from citrus waste using pure water as the solvent has been carried out (133).

An economic and environmentally friendly UAE treatment free of organic solvents performed at room temperature for only 3 min was shown to lead to a naringin-rich flavonoid extract from grapefruit wastes, exhibiting a TPC of 75.3 mg GAE/g (134).

UAE combined with the use of ChCl-glycerol-based DES has also been reported in the case of lemon peels and other agri-food wastes (135).

Remaining in the field of DES application, ChCl-based DES prepared using glycerol and ethylene glycol at different molar ratio have been evaluated as potential solvents for the recovery of polyphenols from orange peels. Optimal conditions were found to be: DES containing 10% w/w of water, a temperature of 60°C, a solid to liquid ratio of 100 g/L, and an extraction time of 100 min (136) (Figure 9).

Recently, the effects of physicochemical properties of DES (viscosity, pH and polarity) for extracting flavonoids from citrus peel waste have been also investigated. Based on the strong linear dependence of extraction yield on polarity, a ternary DES composed of ChCl–levulinic acid–*N*-methyl urea at a molar ratio of 1:1.2:0.8 provided high extraction yields of total flavonoids (137).

Of course, also MAE has been applied as well to citrus processing wastes.

Hesperidin recovery from immature fruit peels of *Citrus unshiu* has been reported using 70% ethanol at 140°C for 8 min, at a 100 g/L solid to solvent ratio. After 24 h storage at 5°C, *ca*. 48 mg/g of hesperidin were collected (138).

Microwave hydrodiffusion and gravity (MHG) technique has been instead applied to mandarin leaves, under RSM optimized conditions involving 275 W microwave power, 2 g mandarin leaf and 45 s. TPC and total flavonoid content (TFC) values of *ca*. 17 mg/g GAE and 1.7 mg/g of catechin equivalents (CE) were determined, which, although lower compared to those obtained by supercritical fluid extraction (SFE), well-correlated with the antioxidant capacity (139).

In a comparative study performed on the residues of industrial processing of fennels, carrots, lemons and tomatoes, MAE has been applied together with maceration and ultrasound assisted extraction (UAE) for the recovery of phenolic compounds. A power of 750 W was used, with a solid to solvent ratio of 40 g/L and a 5 min extraction time; different solvents (methanol, ethanol, water) were used. MAE proved to be particularly effective in the case of carrot wastes, using methanol:water 1:1 v/v as solvent, whereas pure methanol was found to be the best choice for lemon pomace. This latter, in particular, exhibited promising antibacterial activity against *Pseudomonas aeruginosa* and *Clostridium difficile* (140).

### Pomegranate Byproducts

Apparently, UAE seem to be the only green extraction methodology applied to pomegranate wastes, although a combined ultrasound and microwave assisted extraction methodology has been recently reported to be very efficient for the recovery of ellagic acid from fermented pomegranate wastes (141). Ultrasound pretreatment has been reported as an expedient method to significantly improve punicalagin extraction yield from pomegranate peels using a cellulase-based magnetic nanobiocatalyst. This involved suspension of the solid material in 50 mM phosphate buffer (pH 6) (67 g/L solid to liquid ratio) and 37 kHz ultrasound exposure at 50°C for 20 min (142, 143). Pulsed UAE using water as solvent has been also used for the recovery of polyphenols from pomegranate marc (144).

### Apple Byproducts

The superiority of UAE compared to conventional extraction has been proved also in the case of apple pomace. Indeed, in this case, even more efficient than UAE proved to be the ultrasound-assisted micelle-mediated extraction. A 1% water solution of Rokanol B2 was used as solvent, at a 50 g/L solid to solvent ratio. Ultrasound treatment was performed at 50 Hz and 300 W for 30 min. A 7-fold higher TPC was obtained compared to standard UAE with ethanol or water as solvent. Chlorogenic acid, quercetin, and quercetin glyocosides were identified as the main compounds present in the extract (145).

Notably, antioxidant compounds from apple pomace were also efficiently extracted by scCO_2_ (146). In particular SFE was carried out on fresh, oven- and freeze- dried apple pomace varying pressure (20 or 30 mPa) and temperature (45 or 55°C), in absence and presence of ethanol as co-solvent (5%, v/v). The results were compared to those obtained by Soxhlet extraction with ethanol and boiling water maceration. Results showed that scCO_2_ was able to extract polyphenols mainly from the oven and freeze-dried apple pomace, suggesting that the pre-treatment affects the scCO_2_ extract. However, the overall yields were lower when compared to those from conventional solvents methods. The authors justified this unexpected result with the thermal degradation of polyphenols under the working conditions (45–55°C). Concerning the composition, the isolated fractions were rich in quercetin, catechin, myricetin, phlorizin, and phloretin, conferring a high antioxidant activity. Differently, the extract processed by Soxhlet lacked in some polyphenols, accounting for the decrease in the antioxidant activity. Overall, even if the extraction with conventional technique led to higher yields, the SFE process was able to provide an antioxidant enriched fraction.

### Onion Byproducts

Onion wastes represent another important source, together with grape-derived byproducts, of anthocyanins, which have been recovered with other polyphenols by UAE or extraction with DES. The first involved the use of 90% aqueous glycerol as the solvent, with a 11 g/L solid to solvent ratio (147), whereas a higher solid to solvent ratio (33 g/L), 90 min, and 40°C were found to be the best conditions when ChCl/1,2-propanediol/water 1:1:1 molar ratio DES was tested (148). The highest total phenol and flavonoid content was instead obtained with a 50 g/L solid to solvent ratio (148).

In another paper UAE of quercetin from onion wastes has been reported: the optimal extraction conditions were determined to be an ethanol percentage of 59% and extraction temperature of 49°C, yielding a total quercetin content of 11 mg per g of dry weight, whereas pH, solid to solvent ratio and extraction time did not significantly affect the extraction yields (149).

As to the use of DES, other authors investigated the use of eutectic mixtures composed of ChCl as hydrogen bond acceptors with sucrose (4:1), urea (1:2), and sorbitol (3:1) implemented with different water contents for phenolic antioxidant extraction from onion peels. The best results were obtained with ChCl-urea-water 1:2:4 mol/mol/mol, at 60°C, for 120 min, at a solid to liquid ratio of 20 g/L, which led to a TPC comparable to that obtained using 70% aqueous methanol. The experiments were carried out also in a modified domestic microwave oven, with a significant reduction in extraction times (5–25 min) (150).

Different DES consisting of sodium propionate as HBA combined with glycerol and lactic acid have also been analyzed for polyphenol extraction from onion wastes. The best results were obtained with 85% w/w aqueous glycerol/sodium propionate at a molar ratio 8:1, 10 g/L solid to liquid ratio, a temperature of 80°C and a stirring speed of 900 rpm. These conditions provided antioxidant power and polyphenols content comparable to other green solvents (151).

### Carrot Byproducts

UAE apparently represents the only applied green extraction methodology also in the case of carrot wastes. In particular, chlorogenic acids as well as caffeic acid, catechin and epicatechin have been efficiently recovered by RSM optimized UAE of carrot pomace (152). UAE has been described as a powerful technology also for extraction of anthocyanins from black carrot pomace (153).

### Potato Byproducts

Chlorogenic acids are among the main extractable polyphenols from potato byproducts. Both MAE and UAE have been applied to this aim, with the first again allowing for very short extraction times, although requiring higher temperatures and lower sample to solvent ratio. In particular, based on orthogonal array design, MAE was accomplished at 300 W using 60% ethanol as the solvent, at 80°C, for 2 min, with a solid to solvent ratio of 25 g/L, proving to be more efficient than conventional solvent extraction, especially in terms of solvent volumes (154). The RSM-optimized UAE protocol instead involved use of ethanol/water 55/45 v/v in a ultrasound bath (34 kHz frequency) for 35 min at 35°C and a 100 g/L sample to solvent ratio (155).

A DES composed of glycerol and ammonium acetate (molar ratio 3:1) has been also tested for its efficacy for the recovery of phenols from chlorogenic acid rich agri-food solid wastes, including potato peels. The extraction, performed with 80% w/v DES in water, 10 g/L solid to liquid ratio, at 80°C for 3 h and under constant stirring at 600 rpm, demonstrated that the DES was the most efficient in extracting chlorogenic acid derivatives and superior or equally efficient in recovering flavonoids compared to other green solvents (156).

### Tomato Byproducts

A number of papers describe tomato byproducts processing with MAE under different conditions using ethanol-water as the solvent. Under the global optimized conditions, that is 20 min, at 180°C, with 47% ethanol, a solid to solvent ratio of 45 g/L, and 200 W microwave power, an extraction yield of 76% was obtained, with a TPC value of 43.9 mg GAE/g and a TFC of 3.5 mg CE/g. Although the antioxidant power as determined by the ABTS assay was found to be lower compared to commonly used food additives, the optimized tomato waste extract was considered as a sustainable alternative to be used in the fortification and functionalization of food (157). MAE was also found to be the more efficient technique for water extraction of tomato wastes. In particular, extraction was performed at 750 W, for 90 s, with a solid to solvent ratio of 100 g/L. Under these conditions an extraction yield of 16% w/w was achieved, which is higher than those obtained by conventional extraction methods (158). The effects of solvents, temperature and times on MAE of polyphenols from tomato peels have also been recently systematically evaluated (159).

### Lignocellulosic Byproducts

Ferulic acid and its oligomers were the main phenols identified by HPLC-MS following MAE of brewer's spent grain. 0.75% NaOH was used as the solvent and RSM analysis indicated 15 min extraction time, 100°C extraction temperature and a solid to solvent ratio of 50 g/L as the optimal conditions. A 5-fold higher extraction yield (1.3% w/w) of ferulic acid was obtained with MAE compared to conventional extraction techniques, leading to 0.001–0.27% yields (160).

MAE using 20% ethanol in water as the solvent has been described as an efficient methodology also for the recovery of phenolic compounds from spent coffee grounds (161).

**Figure 10:**
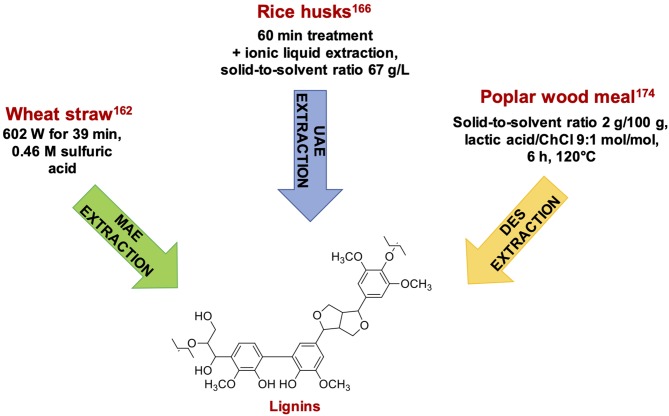
Representative examples of phenolic compounds recovered from lignocellulosic byproducts.

An increase in the wheat straw lignin extraction yield from 3.4 to 11.8% w/w has been also reported, using a microwave radiation power of 602 W for 39 min, and 0.46 M sulfuric acid as the solvent (162) (Figure 10). Another study reported lignin extraction from agri-food wastes by treating the biomass at a 50 g/L solid to liquid ratio in 92% ethanol and 0.32 M sulfuric acid with a microwave power of 250 W for 30 min at 150°C. Under these conditions more than 82% pure lignins were recovered in 35% w/w yield starting from olive kernels (163).

Although no significant improvement was observed in either extraction yields or antioxidant properties when compared to conventional maceration, the advantages offered by MAE in terms of extraction time have been recognized in particular in the case of eucalyptus (164) and chestnut (165) wood industry wastes.

IC_50_ values lower compared to the reference antioxidant butylated hydroxytoluene (BHT) were obtained when MAE was applied to maritime pine (*P. pinaster*) sawdust waste, a byproduct from industry of wood transformation. Both MHG and solvent free microwave extractions were performed, heating the material at 100°C, with a 600 W microwave power, for 40 min. Under these conditions TPC values of *ca*. 75 mg GAE/g extract were obtained, which were higher than those obtained by applying other extraction methodologies (70). A 40% improvement in polyphenol extraction compared to conventional maceration has been reported also when UAE was applied, which apparently involved milder conditions (0.67 W/cm^2^ ultrasonic intensity, 40°C, 43 min) compared to MAE (71).

Other UAE application to lignocellulosic byproducts include use of ionic liquids to extract lignin from rice husks (166) (Figure 10), whereas a phenolic content of 3.1 mg GAE/g of wheat bran has been obtained by UAE using 64% ethanol as solvent, at 60° C, for 25 min (167).

Ultrasound pretreatment of wheat dried distiller's grain, a coproduct from the ethanol production process, has been reported to increase the phenolic compounds extraction yield by *ca*. 14%, as a results of increased pore volume and size (168).

UAE of beech bark at 40 kHz frequency for 20 min, at 65°C, using 70% ethanol as solvent led to a phenolic extract containing 72 mg GAE/g beech bark (169). Polyphenols, particularly phlorizin, have been obtained also from UAE of apple bark using 60% acetone (170).

Ultrasound-assisted enzymatic extraction of protein and antioxidant compounds has been described from sesame bran. The RSM optimized parameters were 836 W ultrasound power, 43°C, 98 min, 9.8 pH value and 1.248 enzyme (alcalase) units /100 g of material, with a solid to solvent ratio of 100 g/L (171).

SFE has apparently not been applied to lignocellulosic byproducts for the recovery of phenolic compounds yet, whereas several applications of DES have been reported in particular not only for lignin extraction but also for lignin processing, being in some cases able to efficiently hydrolyze lignin-carbohydrate bonds in hemicellulose.

Four DES mixtures were prepared using ChCl as HBA and four HBD: acetic acid, lactic acid, levulinic acid and glycerol, in order to solubilize lignin from poplar and Douglas fir wood. At 145°C more than 70% lignin present in poplar and more than 50% present in Douglas fir wood was extracted, with ChCl-lactic acid exhibiting the highest extraction yield (172).

The same DES was found to be the best solvent also in the case of lignin extraction from *Salix matsudana cv. Zhuliu*. After treatment with ChCl-lactic acid 1:10 mol/mol at 120°C for 12 h, the extracted lignin was recovered by precipitation after addition of water and its purity was evaluated, suggesting that the DES not only has a unique capability for the selective extraction of lignin, with a yield of 92%, but is also capable to provide a lignin with high purity degree (95%) (173).

Similar results have been obtained from poplar meal treated with lactic acid/ChCl at 9:1 molar ratio. At 120°C for 6 h an optimal dissolving capacity of 95% has been reached, with a purity of regenerated lignin up to 98.1% (174) (Figure 10).

A facile approach for efficiently cleaving the lignin-carbohydrate bonds using microwave-assisted DES treatment has also been developed. In particular, DES formed by ChCl and oxalic acid dihydrate 1:1 mol/mol was able to solubilize lignin but not microcrystalline cellulose. The extraction was carried out at 80°C, with a microwave power of 800 W and a radiation time of 3 min, which allowed to extract selectively lignin with a high purity (*ca*. 96%) (175).

Other DES have also been evaluated for wood delignification, based on ChCl as HBA and phenol, α-naphthol, resorcinol or maleic acid as HBD, with the aid of ultrasound irradiation. The results showed that all the DES have good solubility properties toward lignin, leading to more than 48% w/w recovery in the case of resorcinol (176).

In another study lignocellulosic biomass fractionation was carried out using different DES, and mixtures of ChCl with oxalic acid and potassium hydroxide allowed to selectively isolate phenols and cellulose, respectively (177).

### Other Fruit and Vegetable Byproducts

A large number of papers report the application of MAE, UAE, SFE, and DES extraction to other agri-food wastes, as summarized in Tables 1–3. Most of these works again highlight the very short extraction times (80 s-37 min) and in some cases the higher yields and antioxidant properties of the extracts obtained with MAE compared to conventional extraction, though sometimes the requirements of higher amounts of solvent has been reported. The higher efficiency compared to traditional extraction also emerges in the case of UAE, which is generally reported to allow the employment of lower temperatures. Also DES generally led to higher extraction yields of polyphenols compared to conventional organic solvents, whereas not much has been reported regarding SFE.

**Table 1:** MAE extraction of phenolic compounds from various agri-food wastes.

**Extraction technique**	**Fruit or vegetable byproduct**	**Extraction conditions**	**Polyphenols extraction yields**	**References**
Microwave assisted extraction (MAE)	Pineapple waste	solid-to-liquid ratio (S/L) 30 g/L, 15 min, 300 W	TPC 12.4 mg GAE/g	(178)
	Banana peel	S/L 28.5 g/L, H_2_O:ethanol 1:1 v/v, 100 s, 380 W	2.2% polyphenols	(179)
		S/L 20 g/L, pH 1, 6 min, 960 W	TPC 53.8 mg GAE/g	(180)
	Xoconostle	S/L 100 g/L, H_2_O, 5.5 min, 297 W	TPC 12.9 mg GAE/g TFC 5.6 mg CE/g	(181)
	*Macadamia tetraphylla*	S/L 50 g/L, H_2_O, 4.5 min, 360 W	TPC 45 mg GAE/g TFC 29 mg rutin equivalents (RE)/g	(182)
	*Sterculia nobilis*	S/L 30 g/L, 41% ethanol,37 min, 67°C, 700 W.	TPC 3.7 mg GAE/g TFC 0.45 mg quercetin equivalents (QE)/g	(183)
	Peanut shells	Irradiation for 2.6 min, followed by incubation with 0.81% w/w cellulase, pH 5.5, 66°C, 120 min.	1.8% polyphenols	(184)
	Apricot kernel skin	S/L 25 g/L, 43% ethanol, 80°C, 20 min, 400 W	TPC 22 mg GAE/g	(185)
	Tobacco waste	S/L 25 g/L, acetone:H_2_O 3:7 v/v, 4 min, 400 W	7.8–12.9 mg CA/g	(186)
	Pequi and jucara waste	S/L 20 g/L, 94% ethanol, 100 s, 670 W	TPC 3.8 mg GAE/g TFC 1.6 mg QE/g	(50)
	Dragon fruit peel	S/L 24 g/L, H_2_O, 45°C, 20 min, 400 W	TPC 58 mg GAE/g	(187)
	Cabbage outer leaves	S/L 100 g/L, ethanol, 5 min, 100 W	TPC 14.9–19.2 mg GAE/g	(188)
	Yarrow dust	S/L 25 g/L, 70% ethanol, 33 s, 170 W.	TPC 238 mg GAE/g TFC 43 mg QE/g	(189)
	Horsetail	S/L 22 g/L, 55% ethanol, 80 s, 170 W.	TPC 162 mg GAE/g	(190)
	Tea residues	230°C, H_2_O, 2 min	74 % polyphenols	(191)
	*Camellia oleifera* meal	S/L 100 g/L, 80% ethanol, 15 min.	TFC 12.8 mg RE/g	(192)

**Table 2:** UAE extraction of phenolic compounds from various agri-food wastes.

**Extraction technique**	**Fruit or vegetable byproduct**	**Extractionconditions**	**Polyphenols extraction yields**	**References**
Ultrasound assisted extraction (UAE)	Walnut green husks	solid-to-liquid ratio (S/L) 50 g/L, 60% ethanol, 60°C, 30 min	TPC 6.9 mg GAE/g	(913)
	*Durio zibethinus M*.	S/L 77 g/L, n-hexane, 5 min, 261 W/cm^2^	TPC 0.7 mg GAE/g	(194)
	Lettuce leaves	S/L 20 g/L, 50–75% ethanol, 120 s, 400 W, 24 kHz	81 μg polyphenols/mL extract	(63)
	Acerola residues	S/L 115 g/L, 46% ethanol, 49 min, 50 kHz, 250 W	TPC 10.7 mg GAE/g TFC 5.6 mg QE/g	(195)
	Capsicum and cabbage waste	S/L 50 g/L, 60% methanol, 37°C, 30 min, 40 kHz.	-	(196)
	Bamboo leaves	S/L 50–100 g/L, 60–90% ethanol, 30–40 min, 150–250 W	TFC 1.5 mg RE/g	(197)
	*Ziziphus mauritiana L*.	S/L 10 g/L, 60% methanol, 30 min	TPC 12.8 mg GAE/g	(198)
	Kudzu roots	S/L 50 g/L, H_2_O/ethanol 2:8 v/v, 80° C, 6 h	7.3 g isoflavones/100 g sample	(199)
	Coconut shell	S/L 20 g/L, 50% ethanol, 30°C, 15 min, 0.487 W/cm^2^	22.4 mg of phenolics/g of sample	(200)
	*Aronia melanocarp*	S/L 25 g/L, 0–50% ethanol, 20–70°C, 0–240 min, 0–100W	TPC >70 mg GAE/g	(201)
	Purple corn cob and husks	S/L 100 g/L, 20 min, 100 W, ethanol/H_2_O/lactic acid 80:19:1	TPC 44-47 mg GAE/g	(202)
	*Euryale ferox*	S/L 37 g/L, 62% ethanol, 40°C, 38 min	TAC 2.8 mg/g	(203)
	Litchi pericarp	Incubation for 90 min with 0.12 mg/mL 1:1 cellulase/pectinase, S/L 67 g/L, 20% ethanol, 50°C, 80 min, 300 W	89.6% procyanidin content	(204)
	*Ginkgo biloba* leaves	S/L 100 g/L, phosphate buffer + 68% ethanol, 8.4 mg cellulase, 40°C, 20 min, 218 W	25.4% flavonoids and 12.4% ginkgolides	(205)
	Star anis residues	S/L 49 g/L, 51 % ethanol, pH 5.3, 45°C, 70 mg/g enzyme, 120 min + 60 min sonication time	14.8% flavonoids	(206)

**Table 3:** UAE, SFE, MHG, and DES extraction of phenolic compounds from various agri-food wastes.

**Extraction technique**	**Fruit or vegetable byproduct**	**Extraction conditions**	**Polyphenols extraction yields**	**References**
Ultrasound assisted extraction (UAE)	Artichoke waste	Solid-to-liquid ratio (S/L) 333 g/L in H_2_O, 60 min, 50W/L	TPC 0.8–1.4 mg GAE/g	(207)
		S/L 100 g/L, 50% ethanol, 25°C, 60 min, 240.	0.02–14.8 mg chlorogenic acid/g	(208)
	Cauliflower waste	S/L 50 g/L, 2 M NaOH, 60°C, 15 min, 37 kHz, 180 W	TPC 7.3 mg GAE/g	(209)
	Tobacco waste	S/L 20-100 g/L, ethanol-H_2_O 60:40–20:80 v/v, 30–70°C, 15–45 min, 37 kHz, 50 W	3.6–804.2 μg/mL of chlorogenic acid 2.34–10.8 μg/mL of caffeic acid 11.6-93.7 μg/mL of rutin	(210)
	Mustard seed meal	S/L 25 g/L, 70% ethanol, 40°C, 30 min, 60 W	TPC 13.8 mg sinapic acid equivalents/g	(211)
Microwave hydrodiffusion and gravity (MHG)	Broccoli waste	43 min, 500 W, under atmospheric pressure, in the absence of solvents	317 μg GAE/mL	(212)
	Sea buckthorn pomace	15 min, 400 W	1147 mg GAE/g	(213)
Supercritical fluid extraction (SFE)	Blueberry waste	Flow rate 0.5 kg/h 5% ethanol + 5% H_2_O as co-solvents, 20 MPa, 40°C	TPC 134 mg GAE/g	(214)
Deep eutectic solvent (DES) extraction	*Ginkgo biloba* leaves	S/L 95 g/L, ChCl/malonic acid 1:2 mol/mol + 55% H_2_O, 65°C, 53 min	22.2 mg proanthocyanidins/g	(215)
	*Moringa oleifera* leaves	S/L 50 g/L, glycerol/sodium acetate 6:1 mol/mol + 20% H_2_O, 50°C, 180 min	TPC 53.8 mg GAE/g TFC 16.5 mg RE/g	(216)
	Peanut roots	S/L 33 g/L, ChCl/1,4-butanediol 1:3 mol/mol + 40% H_2_O, 55°C, 40 min	38.9 mg of resveratrol/kg of sample	(217)
	Rue leaves	S/L 50 g/L, ChCl/citric acid 2:1 mol/mol + 20% H_2_O,30°C, 90 min.	38.2 mg GAE/g	(218)
	Mango waste	S/L 17 g/L, lactic acid/sodium acetate/ H_2_O 3:1:4 mol/mol/mol, 20 min, 436 W	56.2 mg GAE/g	(219)

## Application of Other Sustainable Extraction Methodologies to Agri-food Wastes

**Figure 11:**
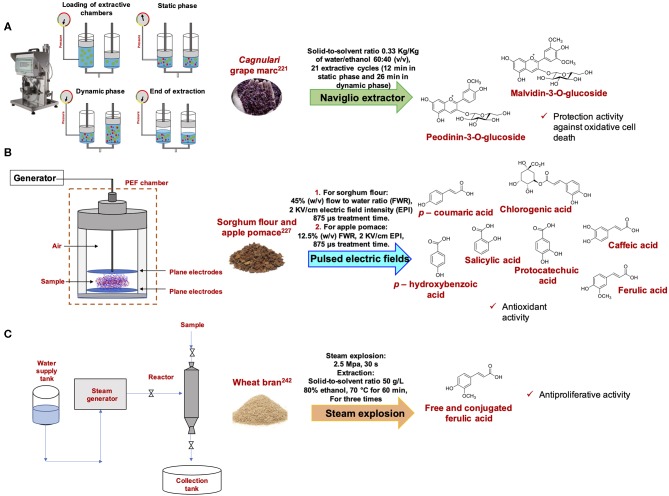
Schematic representation and examples of extraction of bioactive compounds with **(A)** Naviglio Extractor®, **(B)** PEF, and **(C)** steam explosion.

The Naviglio Extractor® is a relatively new solid-liquid extractor that applies the principle that a forced extraction from a solid matrix suspended in a suitable solvent is produced by generating a negative pressure gradient and letting it to go to equilibrium between outside and inside of the solid material (Naviglio's Principle) (Figure 11A). By applying more extractive cycles it is possible to reach the exhaustion of the solid matrix and the extraction of bioactive molecules (220). This new solid–liquid dynamic technology possesses several advantages because it allows to carry out the extraction at room or sub-room temperature thus avoiding thermal stress on thermolabile substances (220). Moreover, the employment of high pressures allows a reduction in the extraction time and a concomitant improvement of the extraction efficacy.

Naviglio Extractor® has been applied to the recovery of phenolic antioxidants from the *Cagnulari* grape marc. The extraction, performed using 21 extractive cycles of 1 min and 25 s each for a total of 38 min using water/ethanol (60:40 v/v) as solvent led to recovery of malvidin, peonidin-3-O-glucoside, malvidin-3-(6-acetyl)-glucoside, and malvidin-3-O-glucoside as the main components of the extract exhibiting a TPC of 4.0 g/L. The extract also revealed a significant ability to inhibit the hydrogen peroxide-induced cell death and reactive oxygen species (ROS) generation (221) (Figure 11A). The solid liquid dynamic Naviglio extraction of vine shoot waste from *Vitis vinifera* Airen variety performed in different conditions provided higher flavonoid and phenolic acid yields in comparison with others solid-liquid extraction methods (222). The vine shoot waste aqueous extract, in particular, stimulated *Lactuca sativa* radicule elongation (223). Naviglio extraction has also been reported for the recovery of polyphenols from grape peels (224).

Another non-thermal processing sustainable technology is based on the use of pulsed electric fields (PEF). This is a novel extraction method which involves the application of microsecond (μs) pulses of high electric field to a material placed between two electrodes (225) (Figure 11B). A classical system for the treatment of pumpable fluids is composed of a PEF generation unit that consists of a high voltage generator and a pulse generator, a treatment chamber, a proper product process system and a set of monitoring and controlling equipment (225, 226). PEF treatment is able to induce a permeabilization of the cytoplasmatic membranes, facilitating the release of intracellular compounds from the cells. PEF increases the extraction rates and yields of different compounds and does not affect the quality of the extracted products.

Phenolic acids such as protocatechuic, cholorogenic, and salicylic acids and salicylic, ferulic, *p*-hydroxybenzoic and caffeic acids were found in high concentrations in PEF treated apple pomace and sorghum flour, respectively. The two optimized conditions, 12.5% w/v solid to water ratio, 2 kV/cm electric field intensity and 500 μs treatment time for apple pomace and 45% w/v solid to water ratio, 2 kV/cm electric field intensity and 875 μs for sorghum flour, provided TPC 37% and 25% higher than those obtained by conventional extraction of apple pomace and sorghum flour, respectively (227) (Figure 11B).

PEF-assisted extraction was found to be a suitable technology to maximize total phenolic and flavonoid yields from canola seed cake under optimized conditions (30 V, 30 Hz, 10% ethanol and 10 s exposure time) (228).

The application of PEF improved the recovery of polyphenols also from cocoa bean shell and coffee silver skin (229), Norway spruce bark (230), and blueberry press cake (231).

PEF pretreatment has been also successfully applied to rapeseed stems and leaves (232), fresh tea leaves (233) and borage leaves (234), leading in all cases to an increase in TPC and antioxidant properties of the extracts.

A PEF pretreatment with an energy input of 300 kJ/kg at 20 kV/cm and a subsequent diffusion step in 20% ethanol and 0.3 M sodium hydroxide allowed to obtain high extraction yield of polyphenols from rehydrated flaxseed hulls (235).

The influence of PEF at different intensity levels (0–7 kV/cm) on pressed orange peels has been also evaluated and the results showed that higher electric field strengths led to an increase in total polyphenol extraction yield and antioxidant activity (236).

Another study proposed a combination of PEF and supplementary aqueous extraction (SAE), which allowed a significant increase of high-added value compound yields and antioxidant capacities of extracts from papaya peels (237). Also in the case of mango peels, the application of two-stage PEF + SAE that included PEF-assisted extraction as the first step and supplementary extraction at 50°C, pH 6, for 3 h as the second step, allowed a noticeable enhancement of TPC (+400%) (238).

Steam explosion is another widely employed and environmentally friendly pretreatment method for vegetable materials. It is based on steam hydrolysis at high temperature (160–280°C), followed by sudden release of high pressure (0.7–4.8 MPa) for relatively short retention time (from several seconds to a few minutes). The treated materials are then discharged through restricted orifices, producing an explosive decompression of biomass (239) (Figure 11C). This results in breakdown of the lignocellulosic structure, hydrolysis of hemicellulose compounds, and depolymerization of the lignin compounds due to rupture of rigid cell wall structure. This technique can therefore be employed as a pretreatment to effectively extract bioactive compounds (240).

Steam explosion and UAE were investigated to develop an effective process for the production of valuable phenolic compounds from sugarcane bagasse lignin. Analysis of the extracts revealed the presence of gallic acid, hydroxybenzoic acid, vanillic acid, p-coumaric acid, ferulic acid, syringic acid, and sinapic acid (241).

Also for wheat bran, the steam explosion treatment at 215°C for 120 s provided free phenolic acid and conjugated phenolic acid yields about 39- and 7-fold higher than those obtained with the untreated sample (242) (Figure 11C).

Finally, high concentrations of hydroxytyrosol and tyrosol were found in olive stones (243) and olive mill solid waste or alperujo (244) after steam explosion pre-treatment.

## Conclusions

The main advantages and disadvantages of the extraction methodologies described in this review are briefly summarized in Table 4. Of course, the choice of one methodology over another is dictated not only by consideration of the advantages or drawbacks, but also and above all by the physicochemical characteristics of the materials and the type of compounds to be extracted. As an example, MAE is not recommended for the recovery of thermolabile compounds, but it can be preferable to UAE if the amount of solvent to be used is a critical factor. Compared to MAE and UAE, much less is apparently reported in the literature for other green extraction methodologies, such as extraction with DES and particularly SFE. It is undoubtedly, however, that these emerging techniques will be more and more exploited in the next future to comply with a total respect of the environment and of the green chemistry principles. Indeed, SFE represents a highly clean, no-solvent technology, allowing to operate at very low temperatures, and it can be expected that the current high equipment costs would be significantly reduced as hand when novel perspectives and applications of this technique will appear in the literature. On the other hand, the added value of DES deriving not only from the low price and biodegradability but also from their ability to induce chemical transformations of agri-food materials (e.g., hemicellulose hydrolysis) resulting in higher extraction yields of bioactive polyphenols will certainly contribute to the enlargement of their application fields.

**Table 4:** Main advantages and disadvantages of the extraction techniques reviewed in this paper.

**Extraction method**	**Advantages**	**Disadvantage**
MAE	• Fast extraction • Low solvent consumption • High extraction yields • Good reproducibility	• High equipment cost • Filtration required • Very poor efficiency for volatile compounds
UAE	• High extraction efficiency • Fast and selective extraction • Low equipment cost • Low operating temperature • Efficient for thermolabile compounds	• Filtration required • Lack of uniformity in the distribution of ultrasound energy • Potential change in the constitutive molecules • Large amount of solvent
SFE	• Fast extraction • Automated system • No filtration required • Possibility to reuse CO_2_ • No use of toxic solvents • Possibility to tune the polarity of scCO_2_ • Possibility to extract thermolabile compounds at low temperatures	• High equipment cost • Elevated pressure required • Risk of volatile compounds losses • Many parameters to optimize
DES	• Low price • Biodegradable • Very low toxicity • Possibility to tune polarity, viscosity and density • High extraction yields	• Filtration is required • High density and/or viscosity

As a general remark, care should be taken concerning the purity of the extracts obtained, since, given the non-selectivity of the green methodologies described, co-extraction of phenolic compounds with compounds that may be toxic, such as emerging pollutants (EPs), could occur. For example, fruit peels usually contain phytosanitary compounds such as herbicides or fungicides, which although present at low concentrations as the result of post-harvest treatments, could accumulate in the extract thus compromising its safety and limiting its possible uses. On this basis, the development of more selective extraction procedures, particularly in the case of SFE which seems not too much susceptible to extensive modulations of the operative conditions e.g., variation of the co-solvent, represents an important challenge to be faced.

## Author Contributions

LP and AN contributed conception and organization of the manuscript. LP, FM, RN, SM, LV, and AN wrote sections of the manuscript. All authors contributed to manuscript revision, read and approved the submitted version.

## Conflict of Interest

The authors declare that the research was conducted in the absence of any commercial or financial relationships that could be construed as a potential conflict of interest.

## Abbreviations

ABTS: 2,2′-azino-bis(3-ethylbenzthiazoline-6-sulfonic acid)
β-CD: β-cyclodextrin
BHT: butylated hydroxytoluene
CE: catechin equivalents
ChCl: choline chloride
DES: deep eutectic solvents
DPPH: 2,2-diphenyl-1-picrylhydrazyl
EP: emerging pollutants
GAE: gallic acid equivalents
FRAP: ferric reducing/antioxidant power
HBA: hydrogen bond acceptor
HBD: hydrogen bond donor
MAE: microwave assisted extraction
MHG: microwave hydrodiffusion and gravity
PEF: pulse electric fields
QE: quercetin equivalents
RE: rutin equivalents
ROS: reactive oxygen species
RSM: response surface methodology
S/L: solid-to-liquid ratio
scCO_2_: supercritical CO_2_
SFE: supercritical fluid extraction
TAC: total anthocyanin content
TAEC: Trolox equivalent antioxidant capacity
TFC: total flavonoid content
TPC: total phenol content
UAE: ultrasound assisted extraction.
